# Absorption of Salicylic Acid

**Published:** 1885-03

**Authors:** 


### ABSORPTION OF SALICYLIC ACID.

From time to time, very interesting and important facts are developed in the Biological Laboratory of the University of Pennsylvania. Dr. N. A. Randolph and Mr. Samuel G. Dixon have recently published a suggestive note on the absorption of salicylic acid (*The Medical News*, Feb. 14th, 1885.)

Salicylic acid, rubbed up in a thin paste with olive oil, was applied to the uninjured skin in seven cases of rheumatism. In each of the seven cases, the presence of the drug in the urine was demonstrated by the ferric chloride test. Existing rheumatic attacks were relieved in six cases; a negative result was obtained in one case.

The constitutional impress of the drug was obtained by its application to the axilla, side of the chest, and the affected joint. From the happy result of the application to the inflamed joint, Dr. Randolph and Mr. Dixon are inclined to believe that the remedy has a specific local effect.

The drug proved an irritant to the skin in but one case.

The advantages of the exhibition of salicylic acid by the skin are numerous and important. The irritant effect of the remedy upon the gastric mucous membrane, and its disagreeable taste, frequently contraindicate administration *per os*.

The subject is worthy of thorough investigation. Quinine has been exhibited by inunction for a long period of time. Absorption, however, is neither so rapid, nor so certain, moreover, the local action of the drug on the skin is of a highly irritant character.

